# Flavonoids as Prospective Neuroprotectants and Their Therapeutic Propensity in Aging Associated Neurological Disorders

**DOI:** 10.3389/fnagi.2019.00155

**Published:** 2019-06-26

**Authors:** Muhammad Ayaz, Abdul Sadiq, Muhammad Junaid, Farhat Ullah, Muhammad Ovais, Ikram Ullah, Jawad Ahmed, Muhammad Shahid

**Affiliations:** ^1^Department of Pharmacy, University of Malakand, Chakdara, Pakistan; ^2^Department of Pharmacy, University of Swabi, Swabi, Pakistan; ^3^University of Chinese Academy of Sciences, Beijing, China; ^4^Key Laboratory for Biomedical Effects of Nanomaterials and Nanosafety, CAS Center for Excellence in Nanoscience, National Center for Nanoscience and Technology, Beijing, China; ^5^Suliman Bin Abdullah Aba-Alkhail Centre for Interdisciplinary Research in Basic Sciences, International Islamic University Islamabad, Islamabad, Pakistan; ^6^Institute of Basic Medical Sciences (IBMS), Khyber Medical University, Peshawar, Pakistan; ^7^Department of Pharmacy, Sarhad University of Science and Information Technology (SUIT), Peshawar, Pakistan

**Keywords:** Alzheimer’s disease, polyphenols, amyloid beta, cholinesterases, antioxidant, signaling pathways and cognition

## Abstract

Modern research has revealed that dietary consumption of flavonoids and flavonoids-rich foods significantly improve cognitive capabilities, inhibit or delay the senescence process and related neurodegenerative disorders including Alzheimer’s disease (AD). The flavonoids rich foods such as green tea, cocoa, blue berry and other foods improve the various states of cognitive dysfunction, AD and dementia-like pathological alterations in different animal models. The mechanisms of flavonoids have been shown to be mediated through the inhibition of cholinesterases including acetylcholinesterase (AChE), and butyrylcholinesterase (BChE), β-secretase (BACE1), free radicals and modulation of signaling pathways, that are implicated in cognitive and neuroprotective functions. Flavonoids interact with various signaling protein pathways like ERK and PI3-kinase/Akt and modulate their actions, thereby leading to beneficial neuroprotective effects. Moreover, they enhance vascular blood flow and instigate neurogenesis particularly in the hippocampus. Flavonoids also hamper the progression of pathological symptoms of neurodegenerative diseases by inhibiting neuronal apoptosis induced by neurotoxic substances including free radicals and β-amyloid proteins (Aβ). All these protective mechanisms contribute to the maintenance of number, quality of neurons and their synaptic connectivity in the brain. Thus flavonoids can thwart the progression of age-related disorders and can be a potential source for the design and development of new drugs effective in cognitive disorders.

## Introduction

Flavonoids represent a diverse group of naturally occurring compounds which are biosynthesized from phenylalanine, and are ubiquitous to green pigments in the plant kingdom (Havsteen, 2002). Flavonoids have a long history of medical use for the treatment of various medical ailments (Rice-Evans and Packer, 2003). Their great diversity, distribution and easy isolation make them a dominant class of therapeutic agents. Flavonoids are the major building blocks for the synthesis of various drugs and may itself be used as natural products, thus play a pivotal role in the domain of drug design and discovery (Havsteen, 1983). Until now, more than 7,000 flavonoids have been reported from natural sources including medicinal plants, vegetables, fruits and wines. Flavonoids have the ability to bind with numerous body proteins and modify the transporters, enzymes, hormones, DNA, chelation of heavy metals and scavenge the free radicals; therefore, possess strong antioxidant properties (Havsteen, 1983; Robak and Gryglewski, 1988; Morel et al., 1993; Cushnie and Lamb, 2005). A myriad number of pharmacological studies have been reported that suggest their usefulness in the management of diabetes mellitus (DM), cancer, cardiovascular diseases, neurological disorders, inflammation and microbial diseases (Middleton et al., 2000; Marder and Paladini, 2002; Galati and O’Brien, 2004; Cushnie and Lamb, 2005).

Recent studies have shown that regular use of flavonoid-rich foodstuffs can effectively enhance cognitive capabilities in humans (Macready et al., 2009; Socci et al., 2017; Bakoyiannis et al., 2019). Additionally, several flavonoids have been reported to restrain the progression of pathologies of Alzheimer’s disease (AD) and this has been stem from their ability to quash the cognitive deficits in numerous normal and transgenic preclinical animal models (Macready et al., 2009; Spencer, 2010b; Bakoyiannis et al., 2019). The beneficial effects of flavonoids rich foods like cocoa, green tea and blue berry can be attributed to the interactions of flavonoids and their metabolites with numerous cellular and molecular targets (Yevchak et al., 2008; Mastroiacovo et al., 2014). For instance, the specific interactions of flavonoids with receptors within the ERK and PI3-kinase/Akt signaling pathways have been reported to augment the expression of neuromodulatory and neuroprotective proteins as well as enhance the number and strength of different types of neurons (Schroeter et al., 2002; Vauzour et al., 2007a; Spencer, 2008). Concomitantly, their beneficial effects on the cerebrovascular system can improve the cognitive performance of individuals *via* an enhancement in blood flow and stimulation of neurogenesis in brain. Several other mechanisms regarding the beneficial use of flavonoids have been recently reported (Spencer, 2009; Spencer et al., 2009). Flavonoids attenuate the initiation and progression of AD-like pathological symptoms and related neurodegenerative disorders (Williams and Spencer, 2012). The possible mechanisms for these effects include the inhibition of neuronal apoptosis induced by neuro-inflammation, oxidative stress, inhibition of key enzymes involved in the fabrication of amyloid plaques and other pathological products (Williams and Spencer, 2012). Flavonoids thus mediate their neuroprotective effects by maintaining the neuronal quality and number in the key brain areas and thus prevent the onset/progression of diseases responsible for the decrease in the cognitive function.

## Methods

Recent scientific literature published in high quality journals were collected using various search engines including Google Scholar, SciFinder, Science Direct, PubMed, Web of Science, EBSCO, Scopus, JSTOR and other web sources. The scientific literature preferably on dietary flavonoids in context to their neuroprotective properties and their mechanism of action were selected. Literature with scientific rigor published up to 2017 was included.

## Flavonoids Distribution in Nature

**Figure 1:**
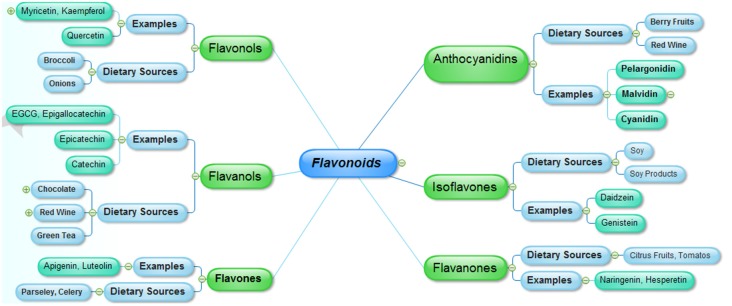
The major classes of flavonoids and their dietary sources.

Flavonoids represent a major group of secondary metabolites which are extensively distributed in nature especially in green plants. Majority of natural flavonoids are pigments, and are usually allied with some vital pharmacological functions. Flavonoids are differentiated from each other on the basis of differences in the aglycon ring structure and state of oxidation/reduction. Moreover, based on the extent of hydroxylation of aglycon, positions of the hydroxyl groups, saturation of pyran ring and differences in the derivatization of the hydroxyl groups are major differentiating features among the various classes of flavonoids. The major nutritional sources of flavonoids include fruits, juices, vegetables, tea, cereals and wines (Manach et al., 2004). Some common flavonoids include quercetin, kaempferol (flavonols), myricetin, predominantly present in the onions, leeks and broccoli, fruits flavones including luteolin and apigenin are abundant in celery and parsley. Other common types of flavonoids include isoflavones (daidzein, genistein), which are naturally distributed in soy and soy products, flavanones including naringenin and hesperetin, present in the citrus fruits and tomatoes. Flavanols, that are represented by epigallocatechin gallate (EGCG), catechin, epicatechin and epigallocatechin are mainly sequestered in the green tea, red wine, and chocolate, whereas, anthocyanidins including malvidin, pelargonidin and cyanidinare are widely distributed in the berry fruits and red wine (Manach et al., 2005; Figure 1).

## Chemistry

**Figure 2:**
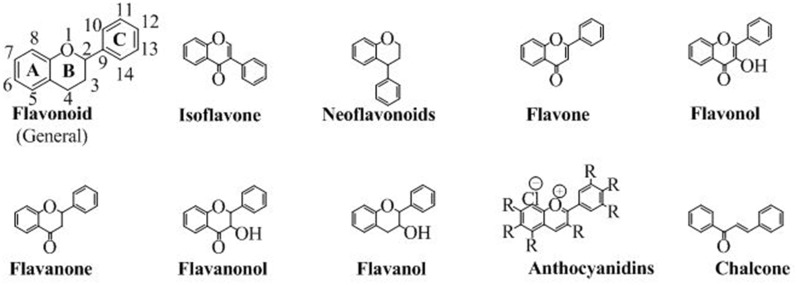
The chemical structures of major classes of flavonoids.

Flavonoids are abundantly present as polyphenols in plants that are the products of secondary metabolites. The basic chemical structure of flavonoids contains two benzene rings (**A** and **C**) connected by a pyran ring **B** (Figure 2). One of the benzene ring (**A**) is fused with the pyran ring while the other benzene ring (**C**) is attached as substituent to the pyran ring. Depending upon the pattern of substitution of benzene rings, and that of substitution, oxidation and saturation of pyran ring, various derivatives of flavonoids can be synthesized that possess unique physicochemical properties and biological activities acceptable for the efficient management of neurodegenerative diseases.

## Classification

Flavonoids are classified into various groups depending on the position at which the benzene ring (**C**) is attached to the pyran and the degree of unsaturation and oxidation of pyran ring. These different flavonoids have a dominant role in various pharmacological activities. Each sub-type is discussed below.

## Isoflavones

**Figure 3:**
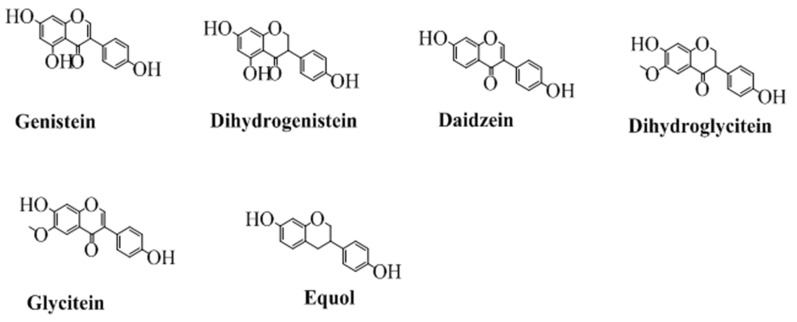
The major isoflavones and their chemical structures.

The class of flavonoids in which the benzene ring (**C**) is attached to the position 3 of the pyran ring is shown in Figure 3. Isoflavone are majorly found in various natural products especially soybean (Wang and Murphy, 1994). Several researchers have also synthesized various derivatives of isoflavone by different synthetic approaches. Wang in 2005 has synthesized various derivatives of isoflavones by Suzuki coupling (Ding and Wang, 2005). Various derivatives of this famous group of easily biodegradable antioxidant have also been synthesized with triazin (Jha et al., 1981). Similarly, utilizing the catalytic approaches, including enzymatic or using a heterogeneous catalyst have been reported for efficient synthesis of isoflavone (Kochs and Grisebach, 1986; Hoshino et al., 1988). The structures of some well-known isoflavones are given in Figure 3.

## Neoflavonoids

In this class of flavonoids, the benzene ring (**C**) is attached to the position 4 of pyran ring. The general structure of neoflavonoids is shown in Figure 2. Neoflavonoids, are naturally occurring heterocyclic compounds, mostly famous for their antidiabetic activity (Donnelly and Boland, 1995). The neoflavonoids consist of neoflavones and neoflavenes. The most prominent source of neoflavonoids is natural but several researchers have also synthesized various analogs. Some natural sources, from which the neoflavonoids are reported, are *Echinop sniveus* (Singh and Pandey, 1990), *Dalbergia odorifera* (Chan et al., 1997), *Nepalese propolis* (Awale et al., 2005), *Polygonum perfoliatum* (Sun and Sneden, 1999) among other important medicinal plants.

## Flavones

**Figure 4:**
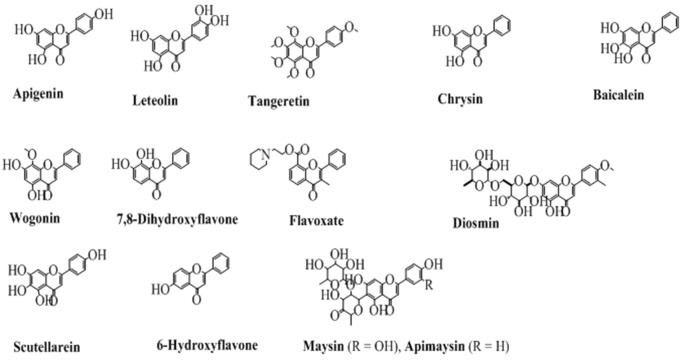
The chemical structures of major flavones derived from natural and synthetic origin.

The flavones contain a double bond on the pyran ring between position 2 and 3, and a carbonyl (ketone) at position 4. Depending upon the taxonomic position of various plants, the flavones contain hydroxyl substituents at both the aromatic rings. Some commonly employed flavones from both the natural and synthetic origin are shown in Figure 4. The history of flavones from natural sources is very common since their synthetic history is also long (Fukui et al., 1968).

## Flavonols

**Figure 5:**
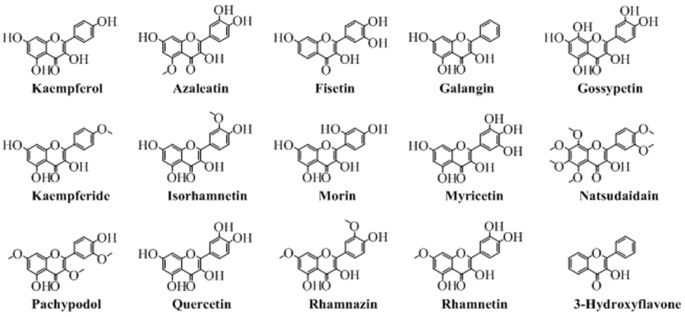
The major naturally occurring flavonols.

Chemically, flavonols are the alcoholic derivatives of flavones. The flavonols differ from the flavones in the hydroxyl group at position 3 of pyran ring. Generally, they can also be called as 3-hydroxyflavones. Mostly, the flavonols are synthesized by synthetic procedures. A very well-known synthesis of flavonols is by oxidation and cyclization of chalcones which ends with 3-hydroxyflavonols. Figure 5 shows the various important flavonols. In some cases, one or more hydrogen of hydroxyl group is replaced by a glucose moiety leading to a flavonol glycoside. As obvious from Figure 5 that pachypodol is not exactly a flavonol but its hydroxyl group is converted into a methoxy group. However, due to its structure resemblance, it can be classified as a derivative of 3-hydroxyflavone, a flavonol.

## Flavanones

**Figure 6:**
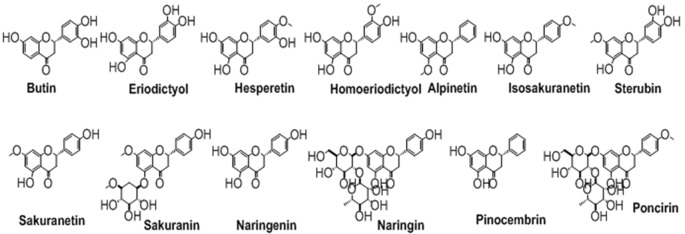
The chemical structures of important isolated flavanones.

The flavanones, saturated flavones, are also known as dihydroflavones. The only difference between flavones and flavanones is the absence of double bond between position 2 and 3. These types of compounds are shown in Figure 6.

## Flavanonols

**Figure 7:**
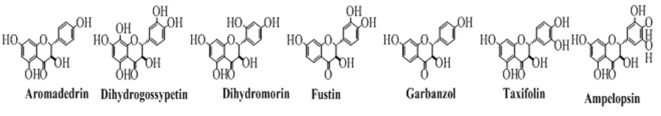
The important members of the flavanonols class of flavonoids.

The flavanonols are the 3-hydroxy flavanones and are also called dihydroflavonols. These are the flavonoids with saturated pyran ring having a hydroxyl group at position 3 and a carbonyl group at position 4. Some common examples of this class of flavonoids are shown in Figure 7.

## Flavanols

**Figure 8:**
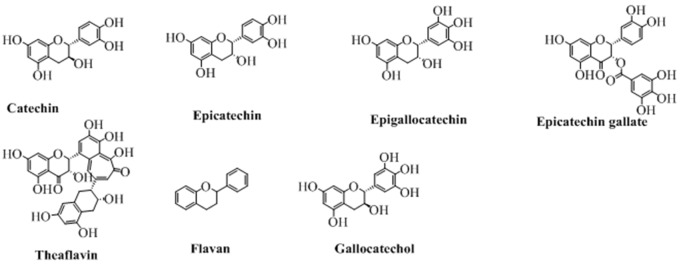
The major flavanols derived from natural sources.

The flavanols, also called flavan-3-ol are the types of flavonoids which lack the carbonyl group at position 4. The pyran ring in these types of compounds is saturated and disubstituted at position 2 and 3. This property of the structure leads to four possible diastereomers of a flavanol. In flavanols, the benzene ring (**C**) is attached to position 2 while the hydroxyl groups at position 3 of pyran ring. The structures of this type of flavonoids are shown in Figure 8. Of these, flavonoids not exactly fit in the definition of flavanol because of a lack of hydroxyl group at position 3. But, still can be categorized under the heading of flavanols as it is structurally similar to other flavanol except the hydroxyl group at position 3.

## Anthocyanidins

They are the only flavonoids which impart color. They are available in the cations form (as chloride salts). They are the salt derivatives of 2-phenylchromenylium (flavylium) cation. This group contains aurantinidin, capensinidin, cyaniding, delphinidin, europinidin, hirsutidin, malvidin, pelargonidin, peonidin, petunidin, pulchellidin and rosinidin. All of them are different from each other on the basis of the attached groups (denoted by R) as shown in Figure 2.

## Chalcones

Although they do not have the pyran ring but are classified as flavonoids because of having a similar synthetic approach to flavonoids. Moreover, in chalcones, the pyran moiety is available as open structure. The open structure has a carbonyl conjugated to a double bond making an α, β-unsaturated ring system, an ideal Michael acceptor for many organic reactions. The structure of chalcone is shown in Figure 2.

## Flavonoids and Alzheimer’S Disease

AD, a neurodegenerative disorder, which is characterized by a gradual memory loss, cognitive dysfunction, imperfection in the routine activities, and a decrease in the intellectual learning process (Sadiq et al., 2015; Ayaz et al., 2017b; Ovais et al., 2018). AD is the most common cause of dementia and affects approximately 5%–8% of individuals over age 65, 15%–20% of individuals over age 75, and 25%–50% of individuals over age 85. It is estimated that 35.6 million people are living with dementia worldwide (Duthey, 2013). Although, the exact etiology of AD is still not known, several mechanistic features including the deficiency of cholinesterases, deposition of β-amyloid plaques, hyperphosphorylation of tau proteins and generation of oxidative stress have been implicated in the development as well as progression of AD (Kamal et al., 2015; Ullah et al., 2016). Due to the diverse nature of these pathological targets, the development of useful anti-AD drugs is still a challenging task for the scientific community. Consequently, multiple targets including the inhibition of key enzyme implicated in AD like acetylcholinesterase (AChE), butyrylcholinesterase (BChE), β-amyloid cleaving enzyme (BACE-1), monoamine oxidase (MAO) and antioxidant agents are currently under investigation as a new therapeutic class of anti-Alzheimer’s agents (Grill and Cummings, 2010; Ahmad et al., 2016; Balducci and Forloni, 2018; Chaudhary et al., 2018).

Currently, only five drugs have been marketed for the management of AD, among them four drugs including galantamine, tacrine, rivastigmine and donepezil are cholinesterase inhibitors whereas, the fifth one is the glutamatergic system modifier called memantine (Ayaz et al., 2015). No anti-amyloid drug is currently clinically available, though several agents are in the different phases of clinical trials (Vassar, 2014). Due to the toxicity associated with the use of currently available drugs and their limited therapeutic effectiveness, the search for new anti-AD drugs is still underway (Ayaz et al., 2014; Ahmad et al., 2015). Consequently, the multi-targeting natural products based pure pharmacological moieties having more bio-safety and promising cognitive enhancing capabilities are among the potential therapeutic agents (Baptista et al., 2014; Bakhtiari et al., 2017; Farooqui, 2017; Khan et al., 2018). Flavonoids including epicatechin-3-gallate, gossypetin, quercetin and myricetin are reported to block β-amyloid, and tau aggregation, scavenge free radicals and sequester metal ions at clinically low concentrations (Ono et al., 2003; Weinreb et al., 2004; Reznichenko et al., 2006; Ansari et al., 2009). Furthermore, xanthone flavonoids have also been reported to scavenge the reactive oxygen species (ROS), inhibit MAO and AChE enzymes (Zhang et al., 2006; Khan et al., 2009; Jayasena et al., 2013). Hence, flavonoids are a promising lead class of compounds for the efficient design and development of multipotent anti-AD drugs.

## Amyloid Precursor Protein (APP), Amyloid Beta (Aβ) and Alzheimer’s Disease

**Figure 9:**
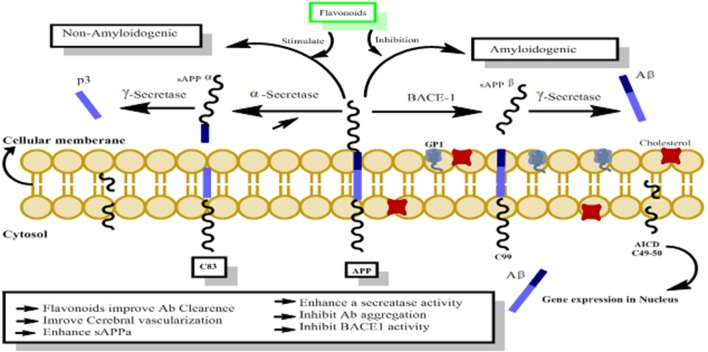
The probable mechanism of flavonoids activating non-amyloidogenic pathway through stimulation of α, γ secretases activities, while inhibiting the neurotoxic amylogenic pathway by inhibition of BACE-1 enzyme.

The amyloid precursor protein (APP) belongs to a group of transmembrane proteins having large extracellular domains (Wasco et al., 1993; Ali et al., 2017; Ayaz et al., 2017a). While members of the APP-like proteins family shares several extracellular domains like E1, E2; however, the amyloid beta (Aβ) domain is unique to the APP protein. APP is produced in the endoplasmic reticulum (ER) and subsequently transported *via* the Golgi apparatus to the trans-Golgi-network (TGN) where APP is found abundantly (Hartmann et al., 1997). APP is transported from TNG by TNG-derived vesicles to the surface of cells where it is enzymatically cleaved by α-secretase, γ-secretases and resulting in the formation of a soluble molecule called sAPPα. 13. This usual process of APP breakdown is non-amylogenic and does not produce Aβ. However, the processing of APP *via* successive actions of beta amyloid cleaving enzyme (BACE-1) and γ-secretase lead to the formation of Aβ as shown in Figure 9 (Nordstedt et al., 1993).

## Pathological Aspects and Drug Targets

### Flavonoids as Cholinesterase Inhibitors

Cholinesterases including AChE, and BChE are involved in the breakdown of acetylcholine (ACh), which is responsible for the impulse transmission across various synapses (Voet and Voet, 1995). Due to the scarcity of ACh in AD, the use of cholinesterase inhibitors is among the useful therapeutic options to maintain the accumulation of neurotransmitter for a long time at the synapse (Bachman et al., 1992). The data regarding the currently available drugs indicate that employing this approach is the most useful target in AD symptomatic therapy, thus streamlining the eventual clinical approval of four drugs (Atta-Ur-Rahman et al., 2004). This approach is also successfully employed in the management of Parkinson’s disease, ataxia and dementia (Ahmad et al., 2003). Owing to the unwanted effects and limited efficacy of the currently available drugs, there is a dire need to develop more safe and effective drugs (Schneider, 2001). Several flavonoids including genistein, kaempferol, apigenin, naringin, quercetin, diosmin, silymarin and silibinin were tested against cholinesterases (AChE, BChE). Among these flavonoids, quercetin was found most active and exhibiting a 76.2% inhibition of AChE. Other compounds including genistein, leteolin and silibinin showed a 65.7, 54.9 and 51.4% inhibitions against BChE, respectively (Orhan et al., 2007). In a published report, Uriarte-Pueyo and Calvo (2011) summarized 128 flavonoids with respect to their AChE inhibitory potentials. Based on their potency as cholinesterase inhibitors, they were considered to be promising therapeutic agents in the development of new anti-Alzheimer drugs.

### Flavonoids as Free Radicals’ Scavengers

Free radicals are generated during the aerobic respiration and are counteracted by the bodily diverse system of antioxidants. When the free radicals are generated in excess, they lead to oxidative stress and thus disturb the functions of different proteins, lipids and essential body elements (Markesbery and Lovell, 2007). Besides their role in several disease processes, free radicals are implicated in the inflammatory damage to neurons and development of AD. The oxidative stress is a key aspect of AD as indicated from the elevated level of oxidative stress markers (Lovell and Markesbery, 2007). Moreover, low concentrations of antioxidants and antioxidant activity have been detected in the plasma of patients diagnosed with AD (Mecocci et al., 2002; Rinaldi et al., 2003). Additionally, the elevated lipid and protein oxidation byproducts were also observed in the transgenic animal models of AD (Resende et al., 2008). The AD pathogenic markers including Aβ and neurofibrillary tangles (NFTs) were also high in animals having oxidative stress, which may suggest that the free radicals are among the initiators of AD (Dumont and Beal, 2011). Nearly all the ROS are generated in the mitochondria (Kowaltowski et al., 2009). In AD patients a deficiency of cytochrome c oxidase leads to the mitochondrial dysfunction and results in the excessive generation of ROS (Müller et al., 2010). Aβ is also considered as mitochondrial poison and is known to initiate the excessive release of free radicals in the presence of metal ions (Butterfield et al., 2007). In this regard, the use of ions like clioquinol is known to exhibit useful effects in transgenic animal models of AD (Grossi et al., 2009).

Activation of glial cells is another hallmark of AD and neurodegenerative disorders (Craft et al., 2005; Balducci and Forloni, 2018). The activation of microglia not only generates pro-inflammatory cytokines but also increases the formation of superoxide anions using NADPH oxidase (NOX). The presence of elevated levels of NOX subunits in the brains of AD and the subsequent improvement of cognitive and cerebrovascular functions after NOX gene removal from the transgenic animals support its potential involvement in the pathogenesis of AD (Park L. et al., 2008). Moreover, in the activated glial cells, inducible nitric oxide synthase (iNOS) sets free the NO, which subsequently reacts with the superoxide and forms peroxinitrite thereby exerting nitrosative stress. Their involvement has been supported by the genetic removal of iNOS which results in the amelioration of gliosis, reduction in Aβ load and phosphorylation of tau proteins in the transgenic animals (Nathan et al., 2005). Catechins and polyphenols of green tea are strong antioxidants, which chelate metal ions and scavenge free radicals (Singh et al., 2008). EGCG prevents oxidative stress-induced DNA damage by transferring an electron to the ROS-induced radical sites (Singh et al., 2008). The green tea suppresses propagation of chain reaction during the lipid peroxidation initiated by the iron ascorbate in the mitochondrial membranes of brain. Among the catechins, EGCG is observed to be the most efficient scavenger (Mandel et al., 2008). EGCG inhibits fibril formation during Aβ aggregation and attenuates the lipid peroxidation as initiated by the Aβ (Choi et al., 2001; Lee et al., 2009). EGCG also inhibits Aβ-induced apoptosis, caspase activity, thus enhancing the survival of hippocampus neurons (Choi et al., 2001).

### Effectiveness in Alzheimer’s Disease and Dementia

The effectiveness of flavonoids in the prevention of AD and cognitive dysfunctions in animal models has been reported, which signify their therapeutic use in the management of neurological disorders. Flavonoids mediate their anti-amyloidogenic effect by targeting key enzymes implicated in the pathological production and accumulation of amyloid plaques (Aβ). Anthocyanin-rich flavonoids found in bilberry and black currant extracts have been recently reported to prevent behavioral abnormalities and alter APP processing in APP/PS1 mouse model of AD (Vepsäläinen et al., 2013). Likewise, chronic therapy with tannic acid using transgenic PSAPP animal model of cerebral amyloidosis has revealed potential amelioration of transgene-mediated deficits in the memory and behavior of animals. A citrus flavonoid nobiletin, has been reported to improve Aβ mediated memory deficits and reduce Aβ load in the hippocampus of transgenic animals (Onozuka et al., 2008). Furthermore, chronic administration of grapes polyphenols leads to improvement in the memory and diminish the level of soluble Aβ oligomers in the brain tissues of Tg2576 animals (Wang et al., 2008). Luteolin, a citrus flavonoid has been shown to decrease the formation of Aβ peptides in APP transgenic neuronal cells and lower the activity of BACE1 (Rezai-Zadeh et al., 2009). Moreover, chronic administration of polyphenol-rich grape seed extracts and curcumin for 9 months inhibit the deposition of Aβ in the brain of AD animals (Rezai-Zadeh et al., 2009).

Numerous studies have demonstrated various beneficial aspects of green tea. Epigallocatechin-3-gallate (EGCG), a green tea polyphenol has been reported to reduce the Aβ load *via* inhibition of APP modulating enzyme (Rezai-Zadeh et al., 2005, 2008). The naturally occurring flavonoids including curcumin and EGCG are reported to restrain Aβ-mediated BACE1 upregulation in the neuronal cultures (Shimmyo et al., 2008). Isorhamnetin has shown a neuroprotective effect against Aβ-induced memory impairment (Asha and Sumathi, 2016). It enhances cognition and memory by uplifting antioxidant defense system, cholinergic signaling, and synaptic plasticity (Ishola et al., 2019). Kaempferol attenuates cognitive deficit through regulating antioxidants and neuro-inflammation (Kouhestani et al., 2018), promotes memory retention and density of hippocampal CA1 neurons (Darbandi et al., 2016). The flavonoid, quercetin has potential therapeutic benefit in AD. Quercetin produces a reduction in plaque burden and mitochondrial dysfunction through the activation of AMPK and may be one of the mechanisms by which quercetin improves cognitive functioning (Wang et al., 2014).

The EGCG-induced increase of non-amyloidogenic APP processing was observed to be carried out through the estrogen receptor-α/phosphoinositide 3-kinase/Ak-transforming based mechanisms. As the post-menopausal depletion of estrogen has been linked to an increased risk of AD development, thus, selective estrogen receptor modulators can be an alternative therapeutic option in the treatment of AD. The use of EGCG mediated estrogen receptor modulation could be an alternative to estrogen-based therapy in the management of this disease (Fernandez et al., 2010). EGCG also produce beneficial neuroprotective effects *via* inhibition of amyloid fibrils sheet rich in Aβ and inhibition of fibrillogenesis. The fibrillogenesis reticence is mediated by direct binding with unfolded polypeptides and inhibition of their conversion to neurotoxic intermediates (Ehrnhoefer et al., 2008). Moreover, EGCG is capable of splitting large size Aβ fibrils to small proteins and thus are not able to aggregate and thereby devoid of any toxic effects (Bieschke et al., 2010). The flavonoid, myricetin has shown potential *in vitro* anti-amyloid activity and thus possesses prospective beneficial effect for neurodegeneration related cognitive disorders (Ono et al., 2003; Hirohata et al., 2007). In general, these reports advocate that some flavonoids have the capability to interrupt fibrillization process of Aβ formation, inhibit a vital enzyme BACE1 implicated in the formation of Aβ, which lead to inhibition of Aβ production. Nevertheless, further studies are required to uncover the neuro-modulating potentials and underlying mechanisms of flavonoids for clinical use.

### Flavonoids as Tau Modifying Agents

**Figure 10:**
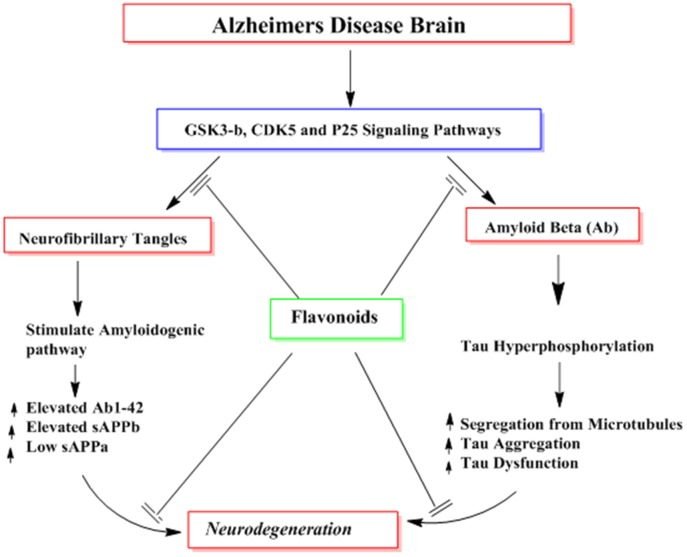
The probable mechanisms of flavonoids in inhibiting different signaling pathways implicated in the formation of neurofibrillary tangles (NFTs) and amyloid plaques (Aβ).

Several reported studies describe the effects of flavonoids in the formation of highly phoshorylated tau proteins, a pathological hallmark of AD (Calcul et al., 2012; Baptista et al., 2014). For instance, myrecetin and epicatechin-5-gallate have been reported to avert heparin-mediated tau formation (Taniguchi et al., 2005). Epicatechin-5-gallate administration in the transgenic animal models of AD has been shown to modulate tau profiles by suppressing the formation of sarkosyl-soluble phosphorylated tau isoforms (Rezai-Zadeh et al., 2008). In other studies using grape seed proanthocyanidin extract (GSPE), tau neuropathology was significantly reduced in animals model of AD *via* inhibition of tau peptide aggregations, its destabilization and its eventual clearance (Pasinetti et al., 2010; Wang et al., 2010). Hyperphosphorylation of tau proteins with subsequent accumulation as NFTs is a major contributor in the cognitive dysfunctions. Several kinases like GSK-3β are known to contribute to the phosphorylation of tau protein and are implicated in the pathogenesis of AD. Flavonoids inhibit the activities of several kinases and thus aid in the prevention of AD. For instance, indirubins restrain the activities of protein kinases including CDK5/p25 and GSK-3β, both of which are implicated in the abnormal phosphorylation of tau proteins observed in the AD patients (Figure 10; Leclerc et al., 2001). Another flavonoid, morin is reported to inhibit the activity of GSK-3β and obstruct GSK-3β-mediated phosphorylation of tau proteins. Morin also diminishes Aβ-mediated phosphorylation of tau proteins and provides protection against Aβ induced cytotoxicity in human neuroblastoma cells. Furthermore, morin therapy has been shown to reduce tau hyperphosphorylation in the hippocampal neurons of transgenic animals (3xTg-AD mice; Gong et al., 2011). Cyanidin 3-O-glucoside (Cy3G) has also afforded a significant protection against cognitive dysfunctions induced by administration of Aβ in animal models which is mediated by modulation of GSK-3β/tau (Qin et al., 2013).

### Neuro-Inflammation and Neurotoxins Modulating Effects

The neurodegenerative outcomes observed in various neurological disorders appear to be elicited by several events like neuro-inflammation, depletion of endogenous antioxidants, glutamatergic excitotoxicity and neurotoxicity mediated by various metabolic products (Jellinger, 2001). Scientific evidence suggest that flavonoids might counteract the underlying mechanisms of neuronal injuries and can hamper the progression of different neurodegenerative disorders (Mandel and Youdim, 2004; Spencer, 2008). Consumption of green tea has been reported to reduce the risk of Parkinson’s disease, attenuate neurodegeneration and ischemic hippocampal injury, which can be attributed to the presence of EGCG (Lee et al., 2000; Weinreb et al., 2004). EGCG is also known to modulate various signaling pathways particularly protein kinase C and PI3-kinase which are implicated in the neuroprotection and reduce the nigral damage by chelating free radicals (Mandel et al., 2005; Weinreb et al., 2009).

Various *in vitro* studies also corroborated the idea that flavonoids prevent the pathological aspects of Parkinson’s disease by inhibiting the formation of endogenous neurotoxin 5-S-cysteinyldopamine (Vauzour et al., 2007b). Moreover, the neuroprotective effects of flavonoids have also been reported in other diseases like Huntington disease, mediated *via* ERK pathway (Maher et al., 2006, 2011). Naringenin, a citrus flavanone has been reported to reduce the neuronal injury *via* inhibition of lipopolysaccharide/interferon-γ-induced glial cells activation and inhibition of p38/STAT-1 pathway (Vafeiadou et al., 2009). Naringenin also inhibits the production of nitric oxide in the activated microglia cells. Blueberry flavonoids also have been shown to attenuate the production of TNF-α, nitric oxide and IL-1β in activated microglia cells (Lau et al., 2007). Other flavonoids including quercetin, wogonin, bacalein and EGCG have been shown to modulate neuro-inflammation and microglial/astrocyte-mediated nitric oxide production (Lee et al., 2003; Chen et al., 2005). All these actions are mediated by transformation of protein, lipid kinase signaling pathways, nitric oxide production, pro-inflammatory transcription factors, downstream regulation of iNOS and cyclooxygenase (COX-2) expression, free radicals scavenging, NOX activation and liberation of cytokine (Jang et al., 2008; Zheng et al., 2008). EGCG and genistein are reported to enhance the production of glutathione *via* PI3-kinase-reliant regulation of nuclear factor erythroid 2–related factor 2 (Nrf2)-induced antioxidant pathway (Hernandez-Montes et al., 2006).

### Flavonoids for Better Cognition

Several studies highlight the beneficial effects of flavonoid-rich foodstuffs’ consumption on cognition (Commenges et al., 2000; Letenneur et al., 2007; Spencer, 2010a). Isoflavones from soy and soy-derived foods have been reported to improve learning and memory possibly by their potential to mimic the activity of estrogens in brain (File et al., 2001). These isoflavones also modulate the neuronal concentrations of ACh and neurotrophic factors including the brain derived neurotrophic factor (BDNF) and nerve growth factor (NGF) in the hippocampus and frontal cortex regions of brain (Pan et al., 1999a,b).

The use of flavonoids rich foods including grapes juice, cocoa and blueberry have shown to possess potential cognition-enhancing effects (Krikorian et al., 2010; Scholey et al., 2010; Shukitt-Hale, 2012). Behavioral evidences suggest that periodic consumption of flavonoids rich fruits like pomegranate, blueberry, grapes, strawberry, as well as pure compounds including quercetin and EGCG are able to improve cognitive performance as indicated from the improvement in the overall scores of memory acquisition, short and long term memory, memory retention and retrieval (Joseph et al., 1999; Hartman et al., 2006). The above mentioned fruits are rich in flavanols and anthocyanins which improve cognitive and spatial working memory deficits in animal models (Joseph et al., 1998; Shukitt-Hale et al., 2009). Additionally, pure EGCG can improve the retention of spatial memory (van Praag et al., 2007). Flavonoids from blueberry also improve the processing of spatial memory *via* its action on the dentate gyrus (DG), which is highly sensitive to the effects of aging (Small et al., 2004; Burke and Barnes, 2006). Blueberry flavonoids have been reported to boost up precursor cells proliferation in the DG of animal models, thus increasing DG neurogenesis and improve cognitive capabilities (Casadesus et al., 2004). However, further characterization of these food supplements, isolation of pure natural compounds and their comparison to the already established flavonoids may provide more useful insights into the memory enhancing properties of dietary flavonoids.

### Flavonoids Interactions With Useful Signaling Pathways

**Figure 11:**
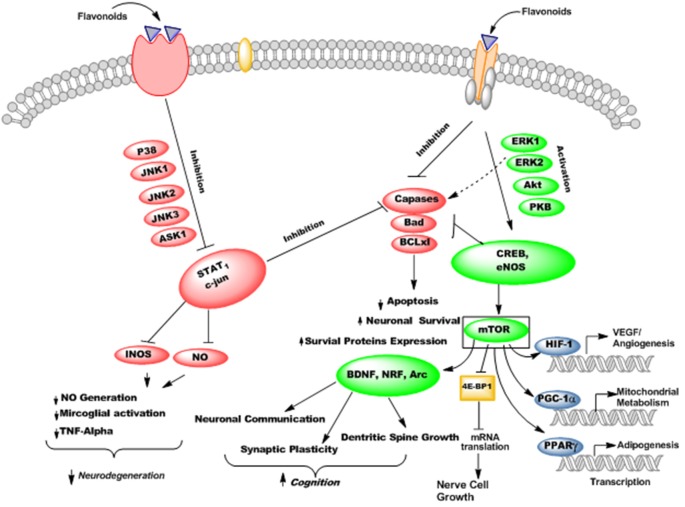
The probable mechanisms of flavonoids stimulating/inhibiting signaling pathways implicated in cognitive performance and neurodegeneration.

Flavonoids are able to preferentially bind with the neuronal receptors including GABA_A_, tyrosine receptor kinase B (TrkB), δ-opioid, estrogen, testosterone, nicotinic and adenosine receptors and mediate the various neuropharmacological actions (Ji et al., 1996; Katavic et al., 2007; Fernandez et al., 2008; Lee et al., 2010). Several reports regarding the beneficial neuroprotective effects of flavonoids and their metabolites *via* interactions with neuronal signaling pathways have been published (Spencer, 2007; Incani et al., 2010). They interact with several protein kinase and lipid kinase signaling pathways like tyrosine kinase, mitogen-activated kinase (MAPK), PI3K/Akt, protein kinase C and nuclear factor κB pathway (Gamet-Payrastre et al., 1999; Schroeter et al., 2001; Incani et al., 2010). When flavonoids bound to these receptors, they may stimulate or inhibit the receptors and thus mediate their actions *via* modulation of gene expression or phosphorylation. Subsequently, they modulate the synaptic protein synthesis, neuronal plasticity and other morphological changes responsible for neurodegenerative disorders and impairment in cognition. For instance, flavonoids and their metabolites have been reported to interact with MAPKs signaling pathways (MEK1 and MEK2 receptors) which result in downstream activation of cAMP response element binding protein (CREB), thus leading to significant changes in synaptic plasticity and memory (Finkbeiner et al., 1997; Impey et al., 1998). Supplementation of flavanols and anthocyanins rich blueberry have been reported to enhance cognitive performance in animals *via* activation of CREB and elevation of BDNF levels in hippocampus (Williams et al., 2008). Furthermore, chronic administration of green tea catechins can reduce the levels of Aβ_1–42_ oligomers, elevate the activities of kinase A/cAMP-response element binding protein (PKA/CREB) pathway and up-regulated the action of synaptic plasticity related proteins in the hippocampus (Li et al., 2009). Moreover, flavonoids stabilize hypoxia-inducible factor-1 (HIF-1) and Nrf2 transcription factors (Park S. S. et al., 2008), activate peroxisome proliferator-activated receptor-γ coactivator-1 (PGC-1α) pathway (Zhang et al., 2010), and act as modulators of peroxisome proliferator-activated receptor gamma (PPAR-γ; Feng et al., 2016). These molecular changes produced by flavonoids may improve AD pathophysiology by protecting neurons against oxidative stress, improve mitochondrial dysfunction, reduce insulin resistance, and thus ameliorate cognitive impairment (Figure 11).

**Figure 12:**
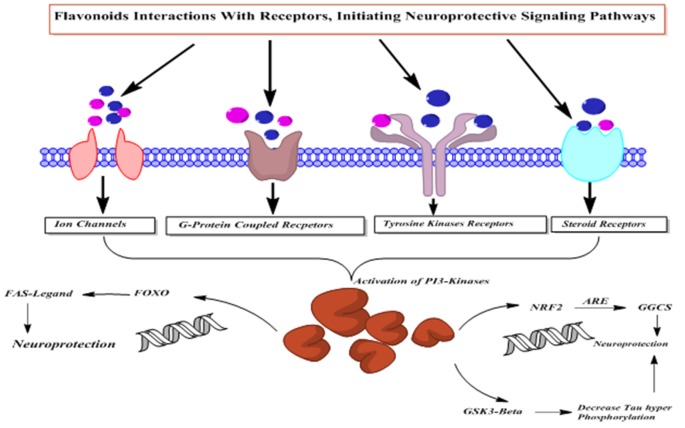
The PI3-kinases activation mediated neuroprotective action of flavonoids.

Flavonoids possess PI3-kinase modulating potentials (Figure 12), by directly interacting with its ATP binding site (Vlahos et al., 1994). Moreover, quercetin and its metabolites inhibit prosurvival Akt/PKB signaling pathways through inhibition of PI3-kinase activity (Spencer et al., 2003). On the contrary, some flavanones like hesperetin activate Akt/PKB signaling pathway and impart prosurvival characteristics in the cortical neurons (Vauzour et al., 2007a). Moreover, epicatechin-5-gallate has been reported to modulate neurotransmission, synaptogenesis and plasticity mediated through stimulation of extracellular signal regulated kinase (ERK), PI3K reliant raise in CREB phosphorylation and upregulation of GluR2 levels in cortical neurons (Schroeter et al., 2007).

In a study, the chronic ingestion of blueberry is reported to increase Akt phosphorylation, activation of downstream mammalian target of rapamycin (mTOR) receptor and increase the content of Arc/Arg3.1 (activity-regulated cytoskeletal-associated protein) in the hippocampus (Williams et al., 2008). As Arc is regulated by BDNF and is important in the long term potentiation (LTP), therefore these changes may be related to the improvement of spatial memory and cognition (Waltereit et al., 2001; Yin et al., 2002). This has been supported by various studies regarding the effects of flavonoids on changes in the neuronal morphologies (van Praag et al., 2007).

### Overview of Mechanisms Underpinning the Therapeutic Effects of Flavonoids in Neurodegeneration

**Figure 13:**
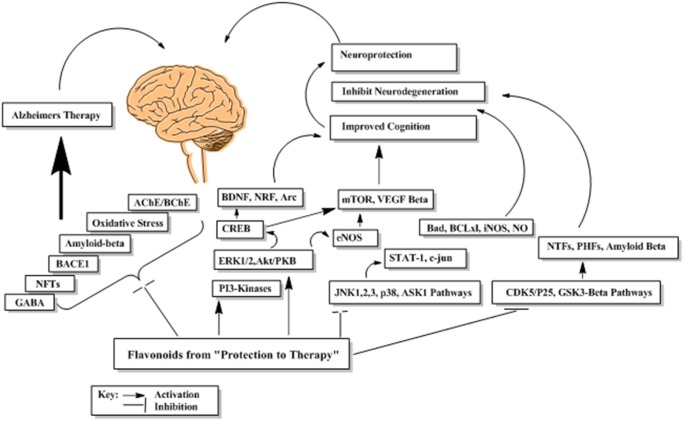
The probable abridged mechanism of flavonoids in enhancing cognition and suppression of neurodegeneration.

Flavonoids by virtue of their low molecular weight, impact multiple cellular targets simultaneously and thus mediate their beneficial neuropharmacological effects in neurodegeneration. Flavonoids interact with several neuronal and glial signaling pathways implicated in neuronal functions and survival (Williams et al., 2004; Spencer, 2010a). They also up-regulate the body antioxidant system and expression of proteins related to neuronal repair and synaptic plasticity (Kong et al., 2000; Eggler et al., 2008). They modulate cerebral blood flow and inhibit neuropathological processing in different regions of brain (Dinges, 2006). The probable mechanism underlying these neuromodulatory properties of flavonoids is shown in Figure 13.

### Toxicological Propensity of Flavonoids

The wide availability of flavonoids and their recent increase consumption by humans has raised important questions regarding the potential toxicity of these dietary components. Although majority of natural products are well tolerated; however, flavonoids and related phytochemicals have been shown to induce neurobehavioral and endocrine disrupting effects (Bugel et al., 2016; Patisaul, 2017). The toxicity of flavonoids is very low in animals. For rats, the LD_50_ has been reported as 2–10 g per animal for most flavonoids. Similar doses in humans are quite unrealistic. As a precaution, doses less than 1 mg per adult per day have been recommended for humans (Galati and O’Brien, 2004). High doses of quercetin over several years has shown to result in the formation of tumors in mice. However, in other long-term studies, no carcinogenicity was found (Dunnick and Halley, 1992). Flavonoids can either inhibit or induce human cytochrome P450 (CYPs) depending upon their structures, concentrations. The interactions of flavonoids with CYP3A4, the predominant human hepatic and intestinal CYP responsible for metabolizing 50% of therapeutic agents is of particular interest. The simultaneous administration of flavonoids and clinically used drugs may cause flavonoid–drug interactions by modulating the pharmacokinetics of certain drugs (Hodek et al., 2002; Galati and O’Brien, 2004).

## Conclusion and Future Directions

The dietary use of flavonoid-rich foodstuffs has the propensity to lessen age-related decline in cognition and may restore memory functions as well as attenuate the development of conditions associated with dementia. The therapeutic importance of natural products in neurodegeneration has been attributed from their various modulatory neuropharmacological properties (Table 1). Further studies are required especially well-designed clinical trials to endorse the clinical effectiveness of flavonoids in neurodegeneration associated clinical signs and symptoms. Moreover, various *in vivo* studies should be designed to obtain a better insight of flavonoids efficacy with regard to their bioavailability, potential toxicities and accumulation at the target sites in the aging brain. For instance, providing a direct link between behavioral responses in test animals/humans to changes in the cortical, and hippocampal areas, the underlying molecular events linked to synaptic plasticity, effects on neuronal stem cells proliferation and changes in the cerebral blood flow will provide guidelines for flavonoids-based dietary applications and subsequent clinical recommendations in neurological disorders. The use of imaging and spectroscopic techniques like MRI and NMR can provide a better understanding of flavonoids-based changes in cerebral blood flow, quantitative changes in neuronal stem cells, progenitor cells and gray matter density along with electrophysiological changes. All these efforts will provide mechanism based links between flavonoids therapy and brain functions and information related to their effective doses. In relation to AD and dementia, it is most important to explore the anti-amyloid and tau modifying effects of flavonoids both in *in vitro* and *in vivo* models. In this regard, tau modifying potentials of flavonoids have been investigated at preliminary level, yet detail studies on destabilization effects of β-amyloid, tau proteins and effects on microglial activation need to be explored. Furthermore, a recommendation regarding the dose/daily intake and duration of therapy must be provided for safe and efficacious results. Molecules which improve the function of CREB are reported to consolidate memory by promoting the gene expression responsible for the synaptic morphology and long term memory. Compounds which activate the function of upstream regulators of CREB, like Akt and ERK are considered to be highly potential memory enhancer drugs. Flavonoids are reported to concentrate in the brain and activate ERK–CREB and Akt–CREB mediated memory and are thus are promising candidates for the development of memory enhancing drugs. Regardless of significant progress in the understanding of flavonoids biology, majority of clinicians mistakenly considered them only as simple antioxidants, which is a major barrier in the development of bioactive flavonoids at the preclinical level. Now it is well known that flavonoids are much more likely to prevent both normal and disease-mediated decline in cognitive functions by modulating cellular and molecular functions of brain. Thus, flavonoids represent a group of vital precursor molecules in the quest to discover new generation of memory-enhancing agents that may be able to counteract and perhaps even quash age-related decline in cognitive functions.

**Table 1:** Summary of the prospective neuropharmacological activities of essential oils and bioactive compounds isolated from medicinal plants.

Flavonoid/s	Source	Study design	Results	Reference
Anthocyanin flavonoids	Bilberry Black currant	APP/PS1 model of AD	↓ Behavioral abnormalities ↓ APP processing	Vepsäläinen et al. (2013)
Nobiletin Naringenin	Citrus Flavonoid/ flavanone	Transgenic AD model ELISA study p38/STAT-1 pathway Lipopolysaccharide/interferon-γ-induced glial cells activation	↓ Aβ load in hippocampus ↓ Aβ-mediated memory deficits ↓ Guanidine-soluble Aβ_1–40_, Aβ_1–42_ ↑ cAMP/protein kinase A response element-binding protein signaling in hippocampal neurons ↓ Aβ induced memory impairment ↓ Neuronal injury ↓ p38/STAT-1 pathway ↓ NO in activated microglia cells	Hernandez-Montes et al. (2006); Onozuka et al. (2008) and Vafeiadou et al. (2009)
Epigalocatechin Galate (EGCG) Genistein	Green Tea	Antioxidant assays Anti-amyloid study Secretases inhibition assays EGCG mediated estrogen receptors (Estrogen receptor-α Phosphoinositide 3-kinase, Ak) modulation. Anti-tau study on AD Transgenic animals Antioxidant studies in neuronal cells	↑ Metal ions chelation ↑ Free radicals scavenging ↓Oxidative stress-induced DNA ↓ Fibril formation during Aβ aggregation ↓ ERK and NF-κB pathways ↓ Aβ induced lipid peroxidation ↓Aβ-induced apoptosis, Capse activity ↑ Neuronal survival ↑ Non-amyloidogenic APP ↓ Fibrillogenesis ↓ Formation of sarkosyl-soluble hosphorylated tau isoforms ↓Risk of Parkinson’s disease ↓ Neurodegeneration ↓Ischemic hippocampal injury ↑ Protein kinase C activity ↑ PI3-kinase activity ↑ Nigral damage *via* scavenging of free radicals ↑ Glutathione	Lee et al. (2000); Lee et al. (2009); Choi et al. (2001); Weinreb et al. (2004); Weinreb et al. (2009); Mandel et al. (2005); Ehrnhoefer et al. (2008); Rezai-Zadeh et al. (2008); Singh et al. (2008) and Fernandez et al. (2010)
Luteolin Curcumin Cyanidin-3-O-glucoside (Cy3G)	Citrus Flavonoid Edible Plants *Curcuma longa* *Hibiscus sabdariffa*	APP Tg neuronal cells AD animal model Tg2576 model	↓ Formation of Aβ peptides ↓ BACE1 activity ↑Soluble Aβ ↓GSK-3 activity ↓ Association of PS1-APP Modulation of GSK-3β/tau ↓ Cognitive dysfunctions	Rezai-Zadeh et al. (2009) and Qin et al. (2013)
Myricetin Indirubins Morin	Vegetables Fruits, Berries Nuts, Tea Edible Plants	AChE inhibition ssay Protein kinases study Aβ induced Cytotoxicity in neuroblastoma cells 3xTg-AD mice	↓ BACE1 activity, Interrupt fibrillization ↓ Heparin-induced tau formation ↓ CDK5/p25, GSK-3β activity ↓ Tau hyperphosphorylation ↓ Aβ-induced cytotoxicity	Leclerc et al. (2001); Ono et al. (2003); Taniguchi et al. (2005) and Hirohata et al. (2007)
Quercetin Wogonin	Fruits, Nuts etc., *Scutellaria baicalensis Georgi*	PI3-kinase inhibitory activity LPS, IFN-γ induced NO production in BV-2 microglia cells Animal model of Inflammation, ischemia Microglia cells study	↓ Akt/PKB signaling pathways ↓ Nitric oxide synthase gene transcription ↓ Neuroinflammation ↓ Nitric oxide production ↓ TNF-alpha, ↓ interleukin-1β ↓ NF-kappa-β activation	Lee et al. (2003); Spencer et al. (2003) and Chen et al. (2005)
Blueberry Flavonoids	Blueberry	Chronic animals study Activated microglia cells	↑ HC Akt phosphorylation ↑ Downstream mTOR activation ↑ Arc/Arg3.1 ↓ TNF-α ↓ NO Production, IL-1β	Lau et al. (2007) and Williams et al. (2008)
Isoflavones Flavanols, Anthocyanins rich food/ Compounds/Extracts	Soy foods Grapes juice, Cocoa Blueberry, Pomegranate	AD animal models Brain estrogen study Behavioral Tasks Memory tasks Locomotor tasks	↑ BDNF ↑NGF ↑CREB ↑ ACh ↑Cognition ↓ Working memory deficits Mimic Brain estrogens activity	Finkbeiner et al. (1997); Impey et al. (1998); Joseph et al. (1998); Pan et al. (1999b); File et al. (2001); Williams et al. (2008); Krikorian et al. (2010); Scholey et al. (2010) and Shukitt-Hale (2012)
127 Flavonoids including, Silibinin Genistein, Apigenin, kaempferol, NaringinQuercetin, Diosmin, Silymarin	Foods Citrus Fruits Natural Products	Cholinesterase inhibition assays	↓AChE activity ↓BChE activity	Orhan et al. (2007) and Uriarte-Pueyo and Calvo (2011)

*↑, Increase/activate; ↓, Decrease/inhibit; HP, hippocampus; AD, Alzheimer’s disease; ACh, Acetylcholine; LPS, Lipopolysaccharide; INF-γ, Interferon-gamma-gamma; BDNF, Brain derived neurotrophic factor; NGF, Nerve growth factor; Arc/Arg3.1, activity-regulated cytoskeletal-associated protein; BACE1, Beta amyloid cleaving enzyme-1; MWM, Morris water Maze; NO, Nitrous Oxide*.

## Author Contributions

MA conceived the idea, carried out literature survey and drafted the manuscript. AS helped in chemistry of flavonoids and corrected the final version of the manuscript. MJ, FU, MO and IU provided useful guidelines, technical support at every step of the manuscript drafting and technical editing. MS and JA performed language and improved technical/scientific aspects of the manuscript, drafted and refined the final version of manuscript. All authors have read and approved the final version of manuscript for publication.

## Conflict of Interest Statement

The authors declare that the research was conducted in the absence of any commercial or financial relationships that could be construed as a potential conflict of interest.

## Abbreviations

AD: Alzheimer disease
NFTs: Neurofibrillary tangles
Aβ: Amyloid beta
iNOS: Inducible nitric oxide synthase
BACE1: Beta amyloid cleaving enzyme
APP: Amyloid precursor protein
ACh: Acetylcholine
AChE: Acetylcholinesterase
BChE: Butyrylcholinesterase
TGN: trans-Golgi-network
EGCG: Epigallocatechin-3-gallate
Cy3G: Cyanidin 3-O-glucoside
ROS: Reactive oxygen species
RNS: Reactive nitrogen species
CAT: catalase
CREB: cAMP response element binding protein
JNK: c-jun N-terminal kinase
p38: protein kinases
BDNF: Brain derived neurotrophic factor
SOD: Superoxide dismutase
COX-2: Cyclooxygenase 2
DG: dentate gyrus
HIF-1: Hypoxia-inducible factor 1
TrkB: Tyrosine receptor kinase beta
MAPKs: Mitogen-activated kinases
mTOR: Mammalian target of rapamycin
Nrf2: Nuclear factor erythroid 2–related factor 2
PGC-1α: Peroxisome proliferator-activated receptor-γ coactivator-1
PPAR-γ: Peroxisome proliferator-activated receptor gamma.
